# A retrospective study of 171 patients with oral lichen planus in the East Bohemia - Czech Republic – single center experience

**DOI:** 10.4317/jced.51784

**Published:** 2014-12-01

**Authors:** Vladimíra Radochová, Ivo Dřízhal, Radovan Slezák

**Affiliations:** 1 M.D, Charles University in Prague, Faculty of Medicine and University Hospital in Hradec Králové, Czech Republic: Department of Dentistry; 2 M.D, PhD, Assoc. Prof, Charles University in Prague, Faculty of Medicine and University Hospital in Hradec Králové, Czech Republic: Department of Dentistry

## Abstract

Objectives: Oral lichen planus is chronic inflammatory disease with a high prevalence in the population. This study describes the epidemiological and clinical characteristics of group of patients with oral lichen planus in the Czech Republic. Material and Methods: Data was taken from the medical records of 171 patients referred to the Oral Medicine Unit at the University Hospital in Hradec Králové with histologically confirmed clinical diagnosis of oral lichen planus in the period 2003 – 2013. The data were retrospectively reviewed. Results: Of the 171 patients, 116 (67.8%) were women and 55 (32.2%) were men. The mean age was 55.2 ± 12.4 years (range of 85.0 – 20.9). The reticular form was the most frequent 93.6% (160 pts.), desquamative gingivitis was 12.9% (22). The buccal mucosa was the site most affected 89.5% (153 pts.). The lesions were asymptomatic in 52 patients (30.4%). Extraoral lesions were observed in 20.5% (35 pts.) of the patients, skin involvement was in 16.4% (28 pts.). Smokers were 29 patients. Local treatment used 116 (67.8%), only 6 patients used systemic short tome corticoid therapy. No evidence between OLP and malignant transformation was observed. Conclusions: This retrospective study show very similar profile and clinical features of the patients with OLP as in other studies.

** Key words:**Oral lichen planus, clinical features, extraoral manifestation.

## Introduction

Oral lichen planus (OLP) is chronic inflammatory disorder that affects the oral mucous membrane. It is a common disease affecting 0.1% to 4% of the population (1-4). The disease usually manifests at the age of 50-70 years and it is very rare in children (5). The etiology and pathogenesis remains unknown but there is overwhelming evidence that cell-mediated immunity is crucial in the pathogenesis. Both antigen-specific and non-specific mechanisms may be involved in the pathogenesis of oral lichen planus. Antigen-specific mechanisms in OLP include antigen presentation by basal keratinocytes and antigen-specific keratinocyte killing by CD8+ cytotoxic T-cells (6). An autoimmune reaction in which CD8+ T lymphocytes attack basal keratinocytes and lead to apoptosis of the cells has been favored. Various potential triggers, e.g. viral or bacterial antigens, metal ions, drugs or physical factors could initiate the autoimmune process (7,8). Genetic involvement in OLP is yet to be determined. OLP can clinically manifest in different forms. Andreasen was the first to classify OLP, and postulated the existence of six different clinical forms (9). Later, this classification was simplified by other authors who basically divided the clinical forms of OLP into reticular, papular, atrophic and erosive lesions or only for red and white forms (5,10). There is often overlap between types, with a combination of reticular, erosive and erythematous lesions. The presentation varies in clinical appearance, with the most lesions being bilateral and located on the buccal mucosa (90%), tongue (30%) and gingiva (13%). Occasionally they can be also found on the lips and palate (4). Isolated lichen planus may be seen in up to 8.6% of patients (11). The patients with OLP may develop lesions that affect the skin, nails or other mucosal surfaces (12). OLP is diagnosed clinically by means of a biopsy for histopathological analysis. The classical microscopic features observed in the oral mucosa include hyperorthokeratosis or hyperparakeratosis, acanthosis, thickening of the spinous layer, liquefaction of the basal layer accompanied by the degeneration of keratocytes and lymphocyte infiltration of the lamina propria (13). The question of treatment is difficult. Treatment should be directed at achieving specific goals after considering the degree of clinical involvement, the predominant clinical type of lesions and the patient´s symptoms. Reticular lesions that are asymptomatic generally require no therapy but only observation for change (14). In general, all treatment should be aimed at eliminating atrophic and ulcerative lesions and alleviating symptoms. In 1978, the World Health Organizations classified OLP as a precancerous lesion, i.e., since then it has been regarded as a generalized process associated with a risk of developing cancer. Although a number of studies have analyzed the malignant transformation of OLP, such malignization remains the subject of controversy (15). To date, most of the more detailed epidemiological and clinical studies of OLP have been undertaken in the other countries but never in the Czech Republic. A general similarity has been confirmed in different populations – including a predilection for women, a mean age about fifty years and the buccal mucosa being the most predilection site. The aim of this study was to undertake a retrospective examination of the general features and clinical presentation of a group of Czech patients with a clinical and histopathological diagnosis of OLP.

## Patient and Methods

The study was approved by local Ethics Committee. The study group comprises 171 patients examined at the Oral Medicine Unit at Department of Dentistry Clinic Charles University in Prague, Faculty of Medicine and University Hospital in Hradec Králové in the Czech Republic in the period 2003-2013 with histologically confirmed clinical diagnosis of OLP according to the diagnostic criteria of World health Organization (WHO) of 1978 modified by van der Meij et al. in 2003 (16). We excluded patients with oral lichenoid contact lesions caused by identifiable cause such as a hypersensitivity reaction to dental restorative materials or patients with lichenoid dysplasia. Only patients with clinical and histological evidence of OLP were included in the study. The following clinical data were obtained from the medical charts: gender, age, clinical presentations of OLP, distributions of the lesions, presence of the symptoms, extraoral manifestations of lichen planus, status of the oral hygiene and periodontal health, presence of systemic diseases, history of the medications, treatment provided (topical corticosteroid in mucosal adhesive paste or as intralesional injection or systemic corticosteroid), adverse effects of treatment, tobacco use. We have also divided the patients into two groups: the first group comprises of the reticular and plaque lesions and second group comprises erosive and erythematous lesions. We compared the groups for possible clinical differences. A descriptive statistical analysis was made using Microsoft Excel 2003 (Microsoft, USA) and MedCalc 9.5.2.0 (MedCalc Software, Belgium). Chi-square and Student t-test were used for comparisons. *P* value < 0.05 was considered statistically significant.

## Results

-Patient´s gender and ethnic origin.

A total of 171 charts of patients with confirmed diagnosis of OLP were retrospectively analyzed, of whom, 116/171 (67.8%) were women and 55/171 (32.2%) were men, giving a female to male ratio of 2.11:1. All of affected patients were white Caucasian.

-Age of onset of oral lichen planus.

**Figure 1:**
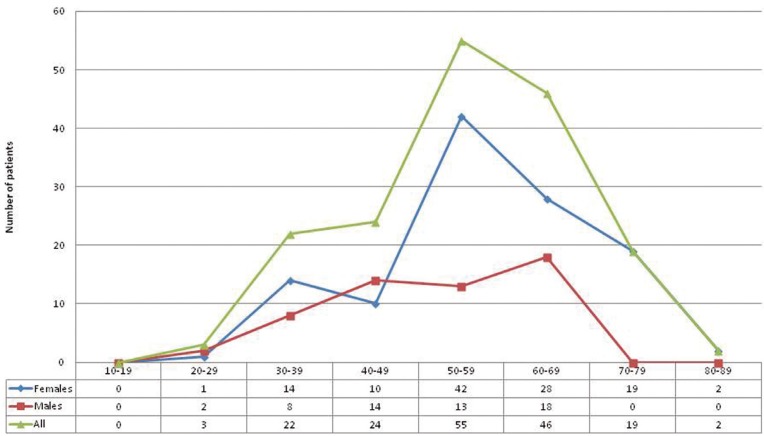
Age distribution of patients with OLP.

 The mean age of the patients at presentations was 55.2 ±12.4 years (mean age for women is 57.0 ± 12.2, for men is 51.2 ± 11.8), with an overall range of 85.0 – 20.9 years. The highest prevalence for women was found in the age group 50-59 years (62/171 pts., 36.2%), for men in the age group 60-69 (56/171 pts., 32.7%) . Age distribution plot is shown in figure 1.

-Systemic diseases, medication, smoking.

**Figure 2:**
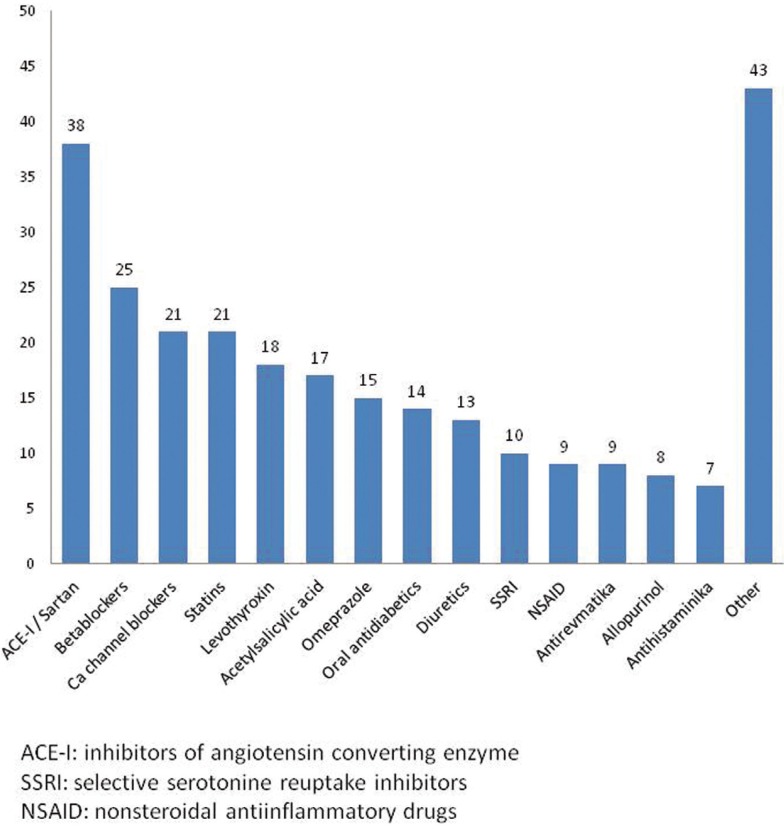
Consumption of drugs by patients with OLP.

Positive history for medication and systemic diseases was found in 117 patients (68.4%). The most prevalent systemic disorders included arterial hypertension (present in 83/171 pts., 48.5% of all patients), thyroid gland disorders (25/171 pts., 14.6%), diabetes mellitus (25/171 pts., 14.6%), hypercholesterolemia (21/171 pts., 12.3%), other cardiovascular diseases (19/171 pts., 11.1%), hypothyroidism (18/171 pts., 10.5%), anxiety/depression (16/171 pts., 9.4%), rheumatological diseases (9/171 pts., 5.2%) and hypeurikemia (8/171 pts., 4.7%). Positive allergy history was in 36/171 patients (21.1%). The medications taken by the patients are shown in figure 2. Most of the patients were non-smokers (142/171 pts., 83%).

-Chief symptoms associated with oral lichen planus.

The presence of the symptoms was reported by the 109/171 patients (63.7%). Severe sustained pain occurred only in 37/171 (21.6%) patients, mild and moderate pain in 72/171 pts. (42.1%). Only 3/171 patients (3.8%) had severe pain in white forms (reticular a plaque lesions), 34/171 patients (37.0%) in red forms (erosive and erythematous lesions). Comparison of erythematous and erosive forms (red lesions) versus the others is shown in  Table 1.

**Table 1:**
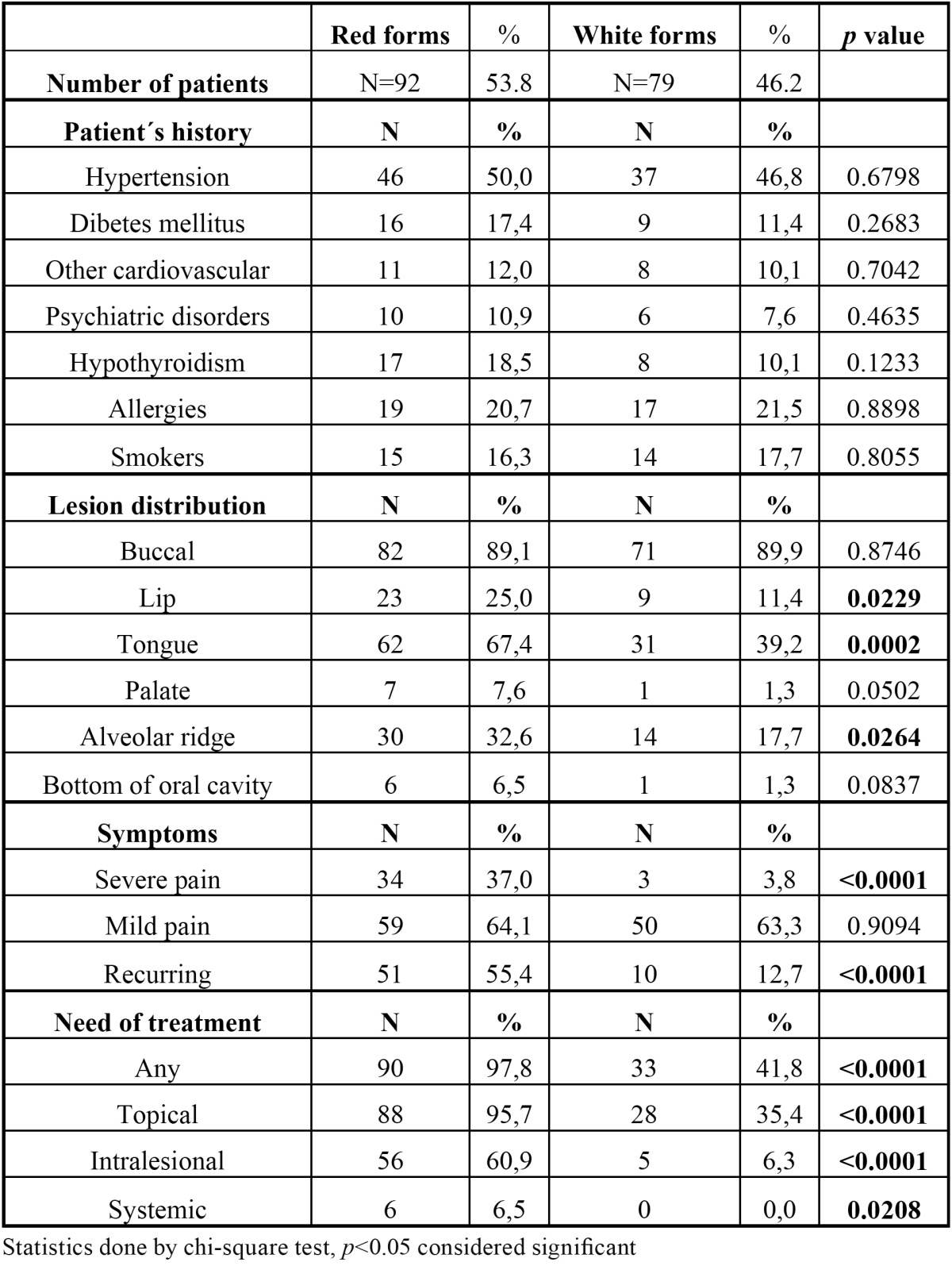
Comparison of patients with red versus white forms of OLP.

-Clinical types of oral lichen planus and distribution.

The most common type was reticular form of the oral lichen planus which was observed in 160/171 (93.5%) patients. 11/171 (6.4%) patients did not show any reticula and were presented only by other clinical forms without reticula. Erosive form was the second most common type with a prevalence of 80/171 (46.8%) pts. Prevalence of the erythematous lesions was 66/171(38.6%) and plaque form was in 56/171 (32.7%) pts. Desquamative gingivitis was present in 22/171 (12.9%) pts. In all cases of desquamation, there was a combination with another form of OLP. 107/171 patients (62.6%) had combination of more types of OLP.

Most of the patients (126/171, 73.7%) exhibited multiple sites of involvement, with the buccal mucosa being the most common location in each clinical form (153/171, 89.5%), followed by the tongue (93/171, 54.4%), gingiva and alveolar mucosa (44/171, 25.7%), lips (32/171, 18.7%). Lesions on the palate (8/171, 4.7%) and floor of the mouth (6/171, 3.5%) were uncommon. No malignant transformation was observed during the observation period.

-Extraoral manifestaion:

Extraoral lesions were observed in 20.5% (35/171 patients) of the patients, skin problems were in 16.4% (28/171 patients). Another 7/171 patients (4%) had genital involvement or the involvement of nails.

-Treatment.

Topical steroids alone were prescribed to 116/171 (67.8%) and in combination with systemic steroids to 6/171 (3.5%) of the patients. 55/171 (32.2%) patients didn´t use the drugs (asymptomatic or the symptoms were very small). In the local treatment we used dexamethasone gel, which patients applied a several times per day to the most symptomatic areas. In 61/171 (35.7%) cases we used combination dexamtehasone gel and depot form of corticosteroid intralesional.

## Discussion

This is as we know the first retrospective analysis of patients with OLP conducted in the east Bohemia. As all retrospective data, it has its major limitations. The biggest bias could potentially be produced by the fact, that our hospital is a tertiary care facility and there is a possibility, that not all the patients from the general practitioners are reported. In general, the results of the present study of OLP are compatible with other previous studies, but we also found some differences which are a matter for discussion. In our study the women were affected in 67.8%, representing the ratio 2.1:1. This number of affected women is similar to the one showed the other studies published by (17). The first manifestation of OLP is usually between 50 and 70 years of age. Our mean age (55.2 years) is similar to that reported by the most of the authors. In the series of 690 patients from UK, reported mean age was 52 years (18). There was a tendency for all types of OLP to occur in male patients at an earlier age than in females. However on the other hand, the highest prevalence in males was between 60-69 years of age. There is also another observation needing attention in our data. We found a small incidence peak in the female group in the age 30-39 years. The precise reasons for this are unknown and probably not of clinical or etiological significance. We think that possible socioeconomic and stress factors may play a role. The coincidence of all systemic diseases is 68.4%. Arterial hypertension is present in 48.5%. It is higher number than for example in Italian study of 808 patients (20% of hypertension) or Spanish study of 550 cases (23% of hypertension) (19,20), but only slightly higher compared to population data in the Czech Republic. For example regarding hypertension there is a general prevalence of hypertension in the Czech Republic about 40% and even higher (60-70%) in patients above the age of 60 (21). Diabetes is also highly prevalent in our population (about 10%) (22) and our data (14.6% of patients with OLP) exceed this number only slightly. Our data show even smaller prevalence of rheumatological diseases, anxiety and depression compared to the previously mentioned studies. We have observed about 10% of patients with hypothyroidism in our group. Recent publication by Robledo-Sierra (23) suggested potential association between OLP and hypothyroidism. The association of these two disorders may not be incidental since both disorders are generally accepted as autoimmune and might share some pathogenetic mechanisms. Extraoral lesions were observed in 20.5 % of the patients. This is comparable to other reported resources such as data from Eisen (24). The intraoral clinical presentation regarding distribution of the lesions is quite the same as reported many times previously (25). All patients have bilateral lesions. Erosive and erythematous OLP (red lesions) represent more than half (53.8%) of the patients. This number of patients is higher compared to series reported previously. In Romanian study by Tovaru (26), there were only 35.8% of red forms and in Brazilian study by Oliveira Alves 41.8% of red forms were reported (27). On the other hand in Turkish study by Gümrü (28), there was a predominance of red forms similar to ours (60 %). As would be expected, the red lesions produce much more clinical problems than the white ones. We could clearly show that red lesions require treatment more frequently than white lesions and also require more often systemic treatment. In our series, the need for topical treatment for serious symptoms was required in 98% of patients with red lesions where only 44% of patients with white lesions required any therapy (*p*<0.0001). Also considering systemic treatment with steroids, all 6 patients who received oral steroid therapy were from the red lesion group (*p*=0.02). The same is true for intralesional application of corticoids. The patients with red lesions were treated with intralesional application in 61% of cases, where only 6% of patients with white lesions required this procedure (*p*<0.0001). There is also an interesting fact regarding the distribution of lesions in the oral cavity. Red lesions are more frequently found on less common places especially palate and alveolar ridge (*p*=0.05, *p*=0.03). Also the recurrence of symptoms after treatment is much higher in red lesions than in white lesions (55% vs. 13%, *p*<0.0001). From this point of view it is clear that occurrence of red lesion is related to clinically much more aggressive behavior and need for further treatment. We have not observed any malignant transformation of OLP in our group of patients. We have to admit that the period of follow up is relatively short and it cannot be excluded that such event might occur in the future. Given the fact, that about 0.5–2% patients with longstanding oral lichen planus may develop a squamous cell carcinoma (29), the periodic observation of these lesions for dysplastic changes remains prudent. However given the very small chance for oral cancer, the real potential for malignant transformation of lichen planus remains controversial. Especially the cost benefit of frequency of following check-up remains an unresolved problem. The fact of carrying a potentially malignant disease must be explained to the patients in great detail, given the fact that only the minority of patient might develop cancer during many years. Having reviewed all the data we came to the conclusion, that Czech population with OLP behaves in very similar pattern as previously published with minor differences of discutable significance.
